# Determining physiotherapy students’ level of knowledge about non-steroidal anti-inflammatory drugs

**DOI:** 10.12669/pjms.42.3.14070

**Published:** 2026-03

**Authors:** Esin Ergonul, Tahir Keskin, Mesut Ergan, Ferdi Baskurt, Zeliha Baskurt, Mehtap Savran

**Affiliations:** 1 Esin Ergonul, MD Associate Professor, Dokuz Eylul University, Faculty of Medicine, Izmir, Turkey; 2 Tahir Keskin Assistant Professor, Mesut Ergan Assistant Professor, Suleyman Demirel University, Faculty of Health Sciences, Physical Therapy and Rehabilitation Department, Isparta, Turkey; 3 Mesut Ergan Assistant Professor, Suleyman Demirel University, Faculty of Health Sciences, Physical Therapy and Rehabilitation Department, Isparta, Turkey; 4 Prof. Ferdi Baskurt, Suleyman Demirel University, Faculty of Health Sciences, Physical Therapy and Rehabilitation Department, Isparta, Turkey; 5 Prof. Zeliha Baskurt, Suleyman Demirel University, Faculty of Health Sciences, Physical Therapy and Rehabilitation Department, Isparta, Turkey; 6 Mehtap Savran, MD Associate Professor, Suleyman Demirel University, Faculty of Medicine, Department of Medical Pharmacology, Isparta, Turkey. Suleyman Demirel University, Faculty of Health Sciences, Physical Therapy and Rehabilitation Department, Isparta, Turkey

**Keywords:** Education, Knowledge, NSAIDs, Physical Therapy, Undergraduate

## Abstract

**Objective:**

This study aimed to evaluate the level and scope of knowledge regarding nonsteroidal anti-inflammatory drugs (NSAIDs) among physiotherapy students.

**Methodology:**

This cross-sectional study was conducted between March and June 2025 and included 175 undergraduate students enrolled in the Department of Physiotherapy and Rehabilitation at a university who had previously completed a pharmacology course. Participants were assessed using a questionnaire that evaluated their knowledge of NSAID indications, contraindications, and adverse effects. In addition, students’ awareness of pharmacology education, current legal regulations, and potential interactions between NSAIDs and physiotherapy modalities was examined.

**Results:**

The majority of students expressed willingness to engage in non-medical prescribing. Participants demonstrated a moderate level of knowledge regarding NSAID indications and contraindications, while the level of knowledge about NSAID-related adverse effects was found to be low. In contrast, students showed a high level of knowledge concerning physiotherapy modalities and drug–physiotherapy interactions.

**Conclusions:**

Although physiotherapy students showed adequate awareness of drug–physiotherapy interactions, their overall knowledge of NSAIDs—particularly regarding adverse effects and clinical safety—was insufficient. Despite their willingness to engage in non-medical prescribing of NSAIDs, these findings highlight the need to strengthen undergraduate physiotherapy curricula with greater emphasis on NSAID-related education.

## INTRODUCTION

Nonsteroidal anti-inflammatory drugs (NSAIDs) are among the most widely used and frequently prescribed medications due to their therapeutic effects, including analgesic, antipyretic, and anti-inflammatory properties.^1^ Given their widespread use in the management of pain and inflammation, NSAIDs are also commonly encountered in physiotherapy practice, where they are used as an adjunct to rehabilitation, particularly in the management of musculoskeletal disorders, acute pain, and inflammatory conditions.^2^-^4^ Despite their well-established benefits, prolonged use of NSAIDs may lead to adverse effects, most notably involving the gastrointestinal and renal systems.^5^ Therefore, healthcare professionals who frequently encounter patients using NSAIDs should have adequate knowledge of their indications, mechanisms of action, adverse effects, and contraindications. Given the high proportion of patients undergoing physiotherapy while using anti-inflammatory medications^2^, it is essential for physiotherapists to be well informed about these drugs.

Within the legal framework of Turkey, physiotherapists are not authorized to prescribe or dispense medications as part of their professional practice. However, the legal status of medication prescribing by physiotherapists varies across countries.^6^–^8^ For example, in the United Kingdom (UK), physiotherapists may become independent prescribers after completing accredited non-medical prescribing programs, which equip them with the competencies required to prescribe medications for the management of musculoskeletal conditions.^9^ In contrast, physiotherapists in many countries do not have legal prescribing authority.^7^ Nevertheless, as healthcare professionals, physiotherapists are frequently consulted by patients, family members, and members of their social environment regarding medication use, and are often asked to provide advice or guidance. Therefore, particularly within multidisciplinary healthcare settings, adequate knowledge of commonly used medications such as NSAIDs is essential for physiotherapists.

Pharmacology education constitutes an integral component of the curriculum in many physiotherapy programs. Within this training, physiotherapy students are educated on drug mechanisms of action, drug–drug and drug–exercise interactions, adverse effects, medication management during exercise, and rehabilitation strategies developed in response to drug-related side effects. This educational content is of particular importance, as physiotherapists encounter clinical situations throughout both their undergraduate education and professional careers that require a sound understanding of commonly used medications, especially NSAIDs. Therefore, assessing students’ knowledge and attitudes toward NSAIDs is essential. However, the adequacy of physiotherapy students’ knowledge regarding NSAIDs remains unclear. Therefore, the present study aimed to assess the knowledge levels and attitudes of physiotherapy students toward NSAIDs. The findings are expected to provide insight into the adequacy of pharmacology education in physiotherapy programs and to inform future improvements in undergraduate curricula.

## METHODOLOGY

This study employed a cross-sectional design to evaluate the level of knowledge regarding NSAIDs among physical therapy students enrolled in the Department of Physical Therapy and Rehabilitation at Süleyman Demirel University. The study was conducted between March and June 2025. All participants were informed about the aims and procedures of the study, and written informed consent was obtained.

### Ethical Approval:

The study was conducted in accordance with the principles of the Declaration of Helsinki. Ethical approval was obtained from the Bingöl University Health Sciences Scientific Research and Publication Ethics Committee prior to data collection (Date: December 26, 2024; Approval No: 24/19).

The study population consisted of 110 third-year and 115 fourth-year undergraduate students enrolled in the Department of Physical Therapy and Rehabilitation, yielding a total of 227 students. No sampling method was applied, as the aim was to include the entire population. Ultimately, 82 third-year students (participation rate: 90.2%) and 93 fourth-year students (participation rate: 80.8%) voluntarily participated in the study.

### Inclusion & Exclusion Criteria:

The inclusion criteria consisted of voluntary participation and having completed the pharmacology course included in the third-year curriculum of the Physical Therapy and Rehabilitation program. Exclusion criteria included being under 18 years of age and refusal to participate in the study after receiving a detailed explanation of the study objectives and methodology.

The questionnaire used in this study was designed using simple and clear language, taking into account the educational background of the target population. It was developed based on relevant literature and reviewed by three academic experts in the fields of physiotherapy and pharmacology to assess content validity. The experts provided qualitative feedback regarding the clarity, relevance, and comprehensiveness of the items, and necessary revisions were made accordingly. No quantitative validation analyses were performed. The questionnaire items administered to the students are provided as Supplementary Material.

The questionnaire consisted of three sections. The first section collected information on the sociodemographic characteristics of the students and their pharmacology education, as well as their knowledge of the legal status of physiotherapists regarding the prescription and recommendation of NSAIDs in accordance with current legislation.^10^,^11^ The second section assessed students’ knowledge of the indications, contraindications, and potential adverse effects of NSAIDs.^12^ The third section examined students’ understanding of the interaction between physiotherapy modalities and NSAIDs and explored the three most common conditions for which NSAIDs are used.^13^

### Statistical analysis:

All data obtained from the participants were analyzed using the IBM SPSS Statistics 20.0 program. Descriptive statistics (percentages, mean, and standard deviation) were utilized to analyze the data. Following this, it was determined that the data obtained at the conclusion of the study demonstrated a parametric distribution.

## RESULTS

A total of 175 physiotherapy students participated in the study. The mean age of the participants was 22.32 ± 1.45 years, 75 (42.85%) students were male and 100 (57.15%) were female. The majority of the students (72.6%) answered “yes” to the question, “*Should physiotherapists be allowed to prescribe NSAIDs*?” Among those who answered affirmatively, the most commonly reported reasons were increased confidence in physiotherapy practice and enhanced professional status (45.7%). Conversely, among students who responded “no,” insufficient education was identified as the primary reason by 56.3% of respondents (Table-I).

**Table-I:** Students’ Opinions Regarding the Prescription of NSAIDs.

Should physiotherapists be permitted to prescribe NSAIDs?			
Yes, n=127 (72.6%)	n(%)	No, n=48(27.4 %)	n(%)
Reasons		Reasons	
- Reducing the workload of doctors	11(8.5)	- Lack of education	27(56.3)
- Increasing the effectiveness of combined therapy	31(24)	- Physiotherapy alone is effective	3(6.3)
- Saving time and costs for patients	7(5.4)	- Physiotherapy losing its traditional role	9(18.8)
- Increasing efficacy of drugs	5(3.9)	- Lack of legality	1(2.1)
- Reduced waiting time of patients	7(5.4)	- It is the duty of doctors to prescribe medicines	8(16.7)
- Increased confidence in physiotherapy, gaining more professional status	59(45.7)		
- Timely drug intervention	9(7)		

**Fig.1:**
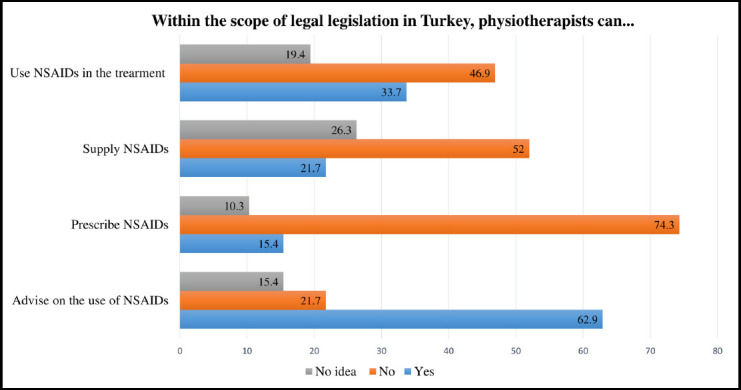
Students’ Opinions on Legal Legislation.

According to the responses, 59 students (33.7%) reported that physiotherapists are permitted to use NSAIDs in patient treatment, while 27 students (15.4%) believed that physiotherapists are authorized to prescribe NSAIDs, and 38 students (21.7%) stated that physiotherapists can supply NSAIDs. In contrast, 110 students (62.9%) indicated that, under current legal regulations, physiotherapists are allowed to provide advice regarding NSAID use (Fig.1).

Furthermore, 131 students (74.9%) reported that any advice given by physiotherapists regarding NSAID use should include appropriate warnings and precautions. Despite this, more than half of the participants (n = 95; 54.3%) stated that they considered their current level of knowledge insufficient to recommend NSAIDs to patients.

A total of 111 students (63.4%) indicated that their undergraduate pharmacological education did not provide adequate knowledge about NSAIDs, while 155 students (88.6%) expressed a desire for more comprehensive NSAID-related instruction at the undergraduate level. Additionally, 138 students (78.9%) indicated a preference for receiving further education on NSAIDs after graduation. Among the students, 74.3%—including those who reported frequent use of drug package inserts as an extracurricular source of information (31.4%)—considered the inclusion of pharmacology education in undergraduate training to be very important.

The students demonstrated a moderate level of knowledge regarding the indications and contraindications of NSAIDs (65.5%). The indications most frequently identified correctly were musculoskeletal pain (82.3%) and inflammation (81.7%). Among contraindications, the highest rates of correct responses were observed for heart disease (74.3%) and history of bleeding (73.3%) (Table-II). In contrast, students’ overall knowledge of NSAID-related adverse effects was low (41.7%). The most frequently identified adverse effects were itching (62.9%) and dizziness (61.1%).

**Table-II:** The students’ answers regarding the indications and contraindications of NSAIDs.

Symptoms	Indication (%)	Contraindication (%)	No Idea (%)
Inflammation	81.7	14.4	2.9
Allergy	26.8	65.1	8.1
Fever	62.2	33.7	4.1
Metastatic bone pain	38.4	52.3	9.3
Ulcer	22	67.3	10.7
Thrombotic disorders	21.5	69.2	9.3
Acute musculoskeletal pain	82.3	11.6	6.1
Heart disease	16.2	74.3	9.5
Dysmenorrhea	27.2	62.7	10.1
Post-operative pain	70.2	22.2	7.6
History of bleeding	16.4	73.3	9.3

**Fig.2:**
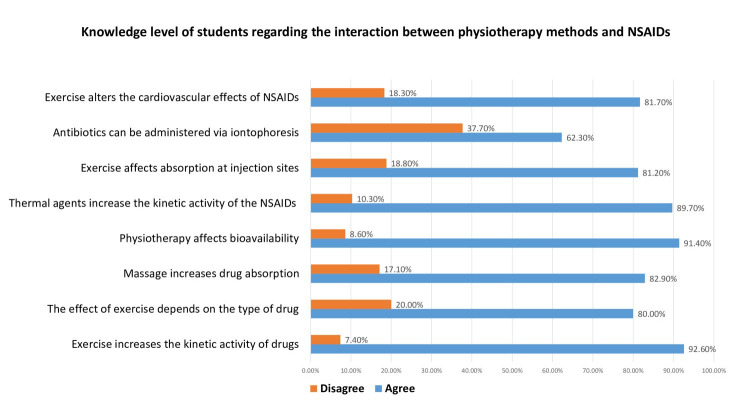
Knowledge level of students regarding the interaction between physiotherapy methods and NSAIDs.

Students demonstrated a high level of knowledge regarding the interactions between physiotherapy modalities and NSAIDs. The highest rate of correct responses (92.6%) was observed for the item related to changes in drug kinetics induced by exercise, whereas the lowest correct response rate (62.3%) concerned the administration of antibiotics via iontophoresis (Fig.2).

The responses provided by the students in response to the question, “Outline three instances in which you consider NSAIDs to be the most frequently employed,” were acute pain (67.4%), rheumatoid arthritis (60%), and osteoarthritis (54.3%).

## DISCUSSION

The present study indicates that physiotherapy students possess a moderate level of overall knowledge regarding nonsteroidal anti-inflammatory drugs (NSAIDs), despite demonstrating an adequate understanding of fundamental pharmacokinetic and pharmacodynamic principles. Notably, the highest level of proficiency was observed in knowledge related to NSAID interactions with other pharmaceutical agents.

In the context of Turkish legislation, physiotherapists are not authorized to prescribe NSAIDs, as this authority and responsibility are exclusively assigned to medical doctors. However, given the nature of physiotherapy, it is a profession group that is frequently encountered with NSAIDs, which are among the most commonly prescribed drugs. Consequently, despite their inability to prescribe these medications to patients, physiotherapists frequently encounter inquiries regarding NSAIDs.

On a global scale, physiotherapists do not have the legal authority to prescribe medications in numerous countries.^14^ However, in countries where the physiotherapy profession is well-developed, such as the UK and Canada, health professionals have the authority to make non-medical prescriptions. Similar developments have been reported in Austria and New Zealand.^13^ In addition, research conducted in Nigeria and several other countries has demonstrated growing interest in this area.^6^,^7^,^10^ In general, it is evident that physiotherapists are also willing to prescribe non-medical prescriptions.^10^,^15^ Overall, the literature indicates that physiotherapists are generally willing to assume non-medical prescribing roles, and patients likewise express support for this expansion of practice.^16^ However, another controversial issue arises in this regard: “Do physiotherapists have sufficient knowledge about non-medical prescribing?” Evidence from earlier studies conducted by Grimmer and Green, respectively, highlights a persistent lack of knowledge among physiotherapists regarding medication prescribing and administration.^8^,^17^ Therefore, it is obvious that the primary issue under discussion is the absence of knowledge. Addressing this gap is essential, and physiotherapy education programs have a critical role in strengthening pharmacological training. The findings of the present study are consistent with the existing literature. Similar to the results reported by Noblet TD et al., physiotherapy students in our sample expressed willingness to adopt non-medical prescribing responsibilities;^18^ however, their current level of knowledge was found to be inadequate. Therefore, pharmacology-related coursework in physiotherapy curricula should be re-evaluated and expanded, particularly with respect to NSAIDs, which physiotherapists frequently encounter in clinical practice.

Pharmacology is an essential component of physiotherapy education, as it supports clinical decision-making, the evaluation of indications and contraindications, and the interpretation of drug interactions throughout the rehabilitation process. In this context, a strong foundation in pharmacology is indispensable for physiotherapist candidates. However, it is also recognized that physiotherapists frequently recommend over-the-counter medications related to their specialty to patients, despite lacking the legal authority to prescribe drugs.^18^ This phenomenon can be attributed to the inherent nature of physiotherapists as health professionals. Physiotherapists often serve as the primary interlocutors for individuals in their care, including patients and their relatives, regarding drug-related inquiries. Furthermore, a significant proportion of patients receiving physiotherapy have multiple co-morbidities, resulting in polypharmacy.^19^ Given the prevalence of NSAIDs as a pharmaceutical intervention in the management of musculoskeletal conditions, it is imperative that physiotherapists possess a comprehensive understanding of these medications.^17^ However, the findings of the present study indicate that physiotherapy students have insufficient knowledge of NSAIDs, with most participants reporting inadequate undergraduate education in this area and expressing a need for enhanced pharmacology training. These findings are consistent with previous reports highlighting physiotherapists’ demand for improved pharmacology education.^5^,^10^,^20^ Addressing this gap may be achieved through expansion of undergraduate pharmacology curricula or the implementation of post-graduate educational programs.

The findings of the present study indicate that students demonstrated a moderate level of understanding regarding the indications and contraindications of NSAIDs, suggesting that they have acquired basic pharmacological knowledge but require further education to manage clinically relevant conditions. Particular attention should be given to gastrointestinal disorders, renal dysfunction, and cardiovascular diseases, which require careful consideration in the context of NSAID use.^21^,^22^ These results are consistent with previous studies conducted among physiotherapists.^5^,^15^,^23^

A more critical concern identified in this study relates to students’ limited awareness of the potential adverse effects associated with NSAID use. This knowledge gap may hinder their ability to appropriately assess risks and provide safe guidance to patients. Given that physiotherapists often function as primary points of contact for patients in many healthcare settings^16^, it is essential that they possess sufficient knowledge and clinical judgment to recognize adverse effects and intervene appropriately when signs or symptoms arise.

Beyond its well-documented benefits, exercise has been shown to influence drug pharmacokinetics and may enhance bioavailability through various physiological mechanisms.^19^ The extent of exercise–drug interactions depends on multiple factors, including the type and duration of exercise as well as the pharmacological properties of the drug.^24^ This multifaceted issue of exercise-drug interactions has the potential to exert a considerable influence on various physiological processes, including bioavailability, drug absorption, distribution, and metabolism. These interactions may affect key pharmacokinetic processes such as absorption, distribution, metabolism, and overall bioavailability. In particular, previous evidence indicates that exercise can enhance drug absorption from intramuscular, subcutaneous, transdermal, and inhalation routes.^24^ In the present study, students demonstrated a high level of knowledge regarding exercise–drug interactions, suggesting an adequate understanding of basic pharmacokinetic principles. This finding indicates that physiotherapy education may effectively convey core concepts related to exercise-induced pharmacokinetic changes; however, such knowledge should be integrated with broader medication safety awareness to support informed clinical decision-making.

### Strength of this study:

A key strength of this study is its comprehensive assessment of physiotherapy students’ knowledge of NSAIDs by integrating pharmacological principles, drug–physiotherapy interactions, and legal considerations within a single framework. By focusing on students who had already completed a formal pharmacology course, the findings provide meaningful insight into existing educational outcomes rather than baseline knowledge. Furthermore, the study addresses an underexplored area in physiotherapy education, particularly the intersection between pharmacology knowledge and non-medical prescribing attitudes. Future studies should adopt multicenter designs, employ formally validated measurement tools, and use longitudinal approaches to examine how targeted curricular enhancements influence knowledge retention, clinical reasoning, and patient safety.

### Limitations:

The primary limitation of this study is its focus on students from a single institution, which may restrict the generalizability of the findings. However, the similarities in pharmacology curricula among physiotherapy programs in Turkey suggest that the results may reflect common educational trends. A secondary limitation of the study is that, although the questionnaire was reviewed by experts for content validity, the lack of a formal pilot test and quantitative validation (e.g., factor analysis or Content Validity Index) is acknowledged as a limitation. Finally, the lack of established external benchmarks for defining adequate knowledge levels in NSAID-related competencies restricts the interpretation of students’ performance relative to accepted standards.

## CONCLUSION

Overall, the findings of this study indicate that despite demonstrating adequate understanding of basic pharmacokinetic and pharmacodynamic principles, physiotherapy students have insufficient level of knowledge regarding NSAIDs when considering all domains collectively, particularly adverse effects and clinical safety. Although physiotherapist candidates expressed willingness to engage in non-medical prescribing of NSAIDs, their current level of knowledge appears insufficient to support such a role. These findings highlight the need for curricular revisions that place greater emphasis on NSAIDs within undergraduate physiotherapy education. In addition, continuous professional development programs focusing on the safe and effective use of NSAIDs are recommended, particularly for physiotherapists working in musculoskeletal rehabilitation, where these medications are most frequently encountered.
